# Evaluation of Chemical Composition, Sun Protection Factor and Antioxidant Activity of Lithuanian Propolis and Its Plant Precursors

**DOI:** 10.3390/plants11243558

**Published:** 2022-12-16

**Authors:** Monika Stanciauskaite, Mindaugas Marksa, Laura Rimkiene, Kristina Ramanauskiene

**Affiliations:** 1 Department of Clinical Pharmacy, Faculty of Pharmacy, Lithuanian University of Health Sciences, Sukileliai Avenue 13, LT-50162 Kaunas, Lithuania; 2 Department of Drug Chemistry, Faculty of Pharmacy, Lithuanian University of Health Sciences, Sukileliai Avenue 13, LT-50162 Kaunas, Lithuania; 3 Department of Analytical & Toxicological Chemistry, Faculty of Pharmacy, Lithuanian University of Health Sciences, Sukileliai Avenue 13, LT-50162 Kaunas, Lithuania

**Keywords:** propolis, poplar, birch, pine, extract, antioxidant activity, UV protection

## Abstract

The growing interest in polyphenols of natural origin and their plant sources encourages the study of their chemical composition and biological activity. Propolis is widely used as a source of phenolic compounds. The aim of this study is to evaluate and compare the chemical composition, antioxidant activity and sun protection factor (SPF) of the ethanolic extracts of the poplar buds, birch buds and pine buds of propolis plant precursors collected in Lithuania. The IC50 concentration of the extracts was evaluated using DPPH and ABTS methods. Extracts of poplar buds, birch buds and propolis showed a lower IC50 concentration by ABTS and DPPH methods compared with pine buds extracts. Poplar buds and propolis extracts showed the highest SPF value, while birch and pine buds extracts showed a lower SPF value. High-performance liquid chromatography (HPLC) analysis results showed that phenolic acids, such as *p*-coumaric acid and cinnamic acid, and flavonoids, such as pinobanksin and pinocembrin, were identified in all the tested extracts. Salicin has been identified only in poplar buds extracts. The results of antioxidant activity showed that propolis poplar and birch buds are a promising source of biologically active polyphenols.

## 1. Introduction

Various plant species and their extracts have been used for medicinal purposes since ancient times [1]. The most commonly studied groups of polyphenols are phenolic acids and flavonoids [2]. Scientists are increasingly focusing on naturally occurring polyphenols and their sources in order to investigate, evaluate and apply extracts of plant materials for therapeutic purposes [3]. Propolis is particularly valued for its various beneficial therapeutic properties, and antioxidant, anti-inflammatory, antibacterial and anti-tumor effects [4]. The therapeutic properties of propolis are due to the presence of various biologically active compounds, such as phenolic acids, flavonoids, sesquiterpenes, lignans, amino acids, vitamins, fatty acids and minerals [5,6]. The flavonoids in propolis are potent antioxidants that can bind free radicals and protect cells from lipid peroxidation [7]. Kim et al. found that propolis inhibited the UV-induced production of (MMP)-1 matrix metalloproteinase in human skin fibroblasts. Propolis reduced UV-induced MMP-1 expression and prevented collagen degradation in human skin tissues [8]. In vitro studies showed that the ethanolic extract of propolis had a protective effect against H_2_O_2_-induced cell death and inhibited the H_2_O_2_-induced decrease in collagen mRNA expression in L929 cells [9]. A study by Gastaldello et al. found that baccharin and coumaric acid isolated from green propolis have anticarcinogenic potential, which may be applicable for the development of new anticancer agents [10].

The process of propolis extraction is a complex one, the chemical composition of which depends on the vegetation of the region where the bees collect propolis. It is important to study in more detail the chemical composition of potential propolis precursors by geographical area and to compare it with the chemical composition of propolis. The chemical composition of propolis is closely related to plant sources, which vary from one geographical area to another [11]. In order to achieve a research-based quality assessment of propolis, it is relevant to investigate the chemical composition and bioactivity of propolis by looking for correlations with the plant precursors of propolis. For example, Pobiega et al. determined the composition of the Polish ethanolic extract of propolis was rich in phenolic acids, such as *p*-coumaric acid, ferulic acid and caffeic acid, and falvonoids, such as pinocembrin, galangin, pinobanksin and pinostrobin [12]. The plant precursors of North American and European propolis are thought to be resins from buds of poplar, aspen, birch, pine, alder, chestnut and oak [13,14,15,16]. In view of the widespread distribution of conifers and birch forests in Lithuania, it is of interest to investigate the chemical composition and biological activity of propolis extracts in comparison with those of birch, pine and poplar buds extracts.

It is known that phenolic compounds can exhibit various properties, such as antioxidant activity and anti-inflammatory activity [17], anti-ageing, antiproliferative, antibacterial and antiviral properties [18,19]. Oxidative stress is responsible for many degenerative diseases, including cancer and cardiovascular diseases [20,21]. Oxidative stress is associated with a high production of reactive oxygen species (ROS) in the cell, exceeding the cell’s ability to remove them efficiently, which can cause damage to DNA, proteins or lipids within the cell [22,23,24]. Research has shown that polyphenols can protect against UV radiation exposure in a variety of ways. Excessive exposure to UV radiation can cause adverse reactions and damage the cell’s DNA [25,26]. Natural polyphenols are often yellow, red or purple pigments that are able to absorb UV radiation [27]. Various clinical and in vitro studies have shown that exposure to UV radiation may be directly responsible for various skin diseases, skin ageing, dry skin, vasodilation, melanoma and skin tumors [27]. Phenolic compounds are characterized by their absorption spectrum, where UV radiation is filtered out, thereby reducing the penetration of harmful UV rays into the skin, oxidative stress and the damaging effects on DNA [26,28]. Protection against the adverse effects of UV radiation from the sun can be achieved by the use of natural polyphenol products, which not only have antioxidant and anti-inflammatory properties but also photoprotective properties [29,30].

Propolis and its plant precursors are potential candidates as active ingredients in skin care and pharmaceutical formulations for the protective effects of solar UV radiation. The photoprotective properties of Lithuanian propolis and propolis plant precursors have not been investigated so far. The results of this study are relevant for the application of propolis in UV-protective products. The aim of our planned study is to compare the chemical composition of ethanolic extracts of propolis collected in Lithuania with its plant precursors in balsam poplar buds (*Populus balsamifera* L.), birch buds (*Betula pendula* L.) and scots pine buds (*Pinus sylvestris* L.) to evaluate their antioxidant and photoprotective properties.

## 2. Results

### 2.1. Evaluation of the Total Content of Phenolic Compounds

**Figure 1:**
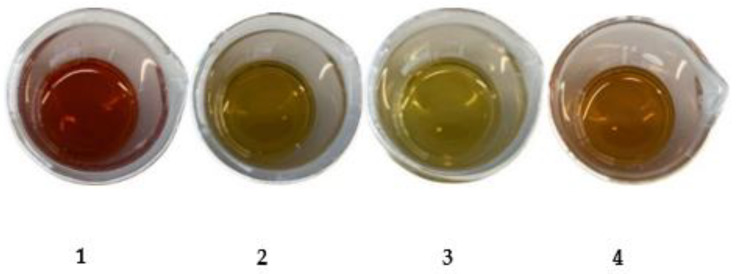
Physical appearance of poplar (1), birch (2), pine (3) buds and propolis (4) extracts.

The ethanolic extract of pine buds had the lowest content of phenolic compounds: 53.93 ± 3.24 mg CAE/g. The highest phenolic compounds content was observed in the balsam poplar buds ethanolic extract: 230.42 ± 14.83 mg CAE/g. A statistically significant difference (*p* < 0.05) was found between the pine buds extract and all the other extracts tested. There was no statistically significant difference (*p* > 0.05) in total phenolic compounds between the balsam poplar buds and propolis extracts. The results are presented in Table 1 and the appearance of the extracts presented in Figure 1.

### 2.2. Analysis of Active Compounds by HPLC Analysis

**Figure 2:**
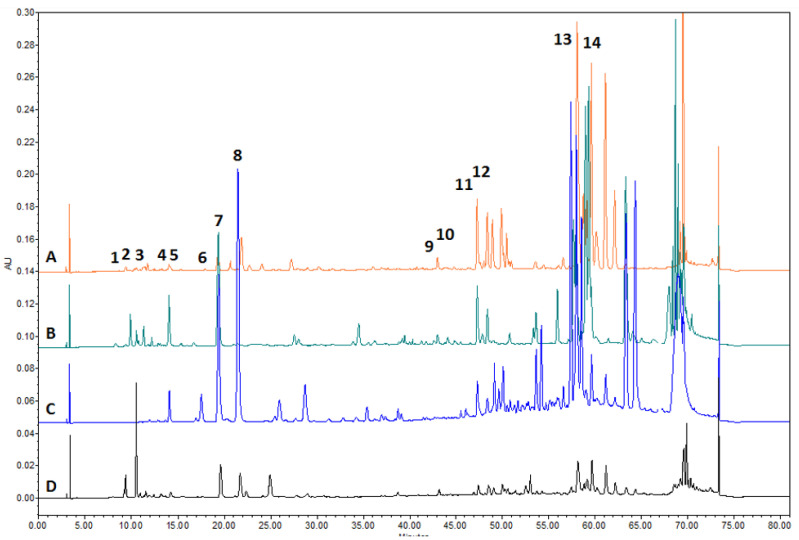
Chromatogram of A—birch buds, B—poplar buds, C—propolis and D—pine buds extracts. 1—salicin, 2—neochlorogenic acid, 3—chlorogenic acid, 4—vanillic acid, 5—caffeic acid, 6—vanillin, 7—*P*-coumaric acid, 8—ferulic acid, 9—cinnamic acid, 10—quercetin, 11—pinobanksin, 12—apigenin, 13—kaempferol, 14—pinocembrin.

The chemical composition of the extracts produced was evaluated by HPLC (Table 2, Figure 2). The balsam poplar buds extracts were dominated by *p*-coumaric acid 12.696 ± 0.366 mg/g, cinnamic acid 8.866 ± 0.167 mg/g and galangin 6.396 ± 0.110 mg/g. These predominant compounds account for about 40%, 28% and 20% of the total identified compounds, respectively. Salicin was found in the poplar buds extract—0.556 ± 0.046 mg/g (Figure 2). In the birch buds extract, the predominant compound identified was pinocembrin—4.940 ± 0.125 mg/g, which accounts for about 70% of the total compounds identified. Additionally, identified in the birch buds extract was *p*-coumaric acid, which was found at 1.167 ± 0.078 mg/g, and apigenin, which was found at 1.030 ± 0.065 mg/g. In propolis, the predominant compounds identified were *p*-coumaric acid 15.776 ± 0.410 mg/g, ferulic acid 7.479 ± 0.227 mg/g and vanillin 4.980 ± 0.163 mg/g, which accounted for about 52%, 25% and 17% of the total identified compounds, respectively. Pinocembrin, ferulic acid and *p*-coumaric acid were detected in pine buds extract but in significantly lower amounts of compounds compared with poplar buds, birch buds and propolis extracts.

### 2.3. Antioxidant Activity of DPPH and ABTS In Vitro

**Figure 3:**
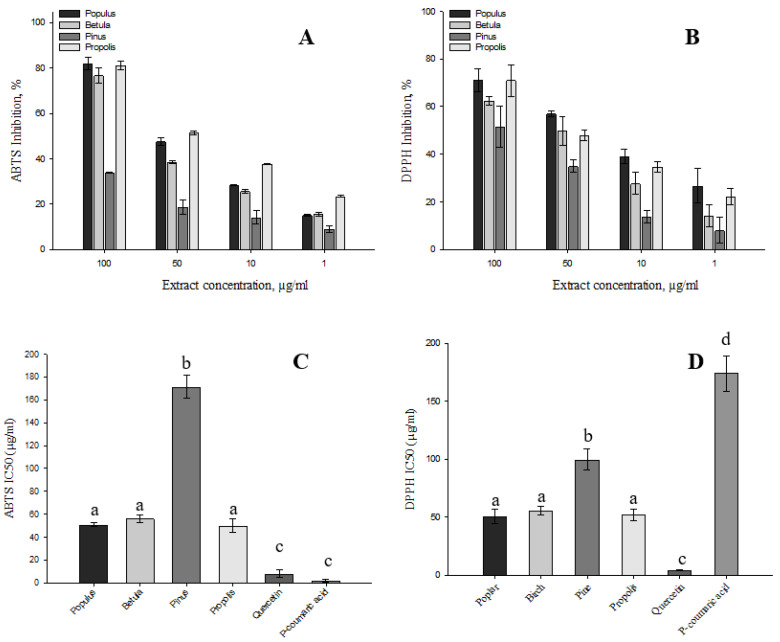
Antioxidant activity of poplar, birch, pine buds and propolis extracts. Subgraph (**A**)—ABTS inhibition % of extracts (1 µg CAE/mL to 100 µg CAE/mL); subgraph (**B**)—DPPH inhibition % of extracts (1 µg CAE/mL to 100 µg CAE/mL); subgraph (**C**)—IC50_ABTS_ concentration; subgraph (**D**)—IC50_DPPH_ concentration. Subscripts of different letters indicate statistically significant differences between subjects (*p* < 0.05).

To assess the antioxidant activity of the extracts, solutions of the extracts were made at different concentrations ranging from 100 µg CAE/mL to 1 µg CAE/mL (Figure 3). The IC50 of the extracts and standards (*p*-coumaric acid, quercetin) evaluated by ABTS showed a statistically significantly higher IC50 concentrations of the tested extracts compared with *p*-coumaric acid and quercetin (*p* < 0.05). The IC50_ABTS_ of all tested extracts ranged from 49.92 ± 6.44 µg CAE/mL to 171.29 ± 10.01 µg CAE/mL. The lowest inhibitory concentrations were observed for the extracts of propolis, poplar buds and birch buds. There was no statistically significant difference in IC50_ABTS_ concentrations between these extracts (*p* > 0.05). When antioxidant activity was evaluated by DPPH, the pine buds extract had the highest inhibitory concentration of 99.53 ± 9.07 µg CAE/mL. The lowest inhibitory concentration of the extracts tested was the poplar buds extract with 50.64 ± 6.49 µg CAE/mL. The IC50_DPPH_ concentration of propolis was found to be 51.92 ± 4.95 µg CAE/mL. There was no statistically significant difference (*p* > 0.05) in IC50_DPPH_ concentrations between the poplar buds and propolis extracts. A statistically significantly higher concentration of IC50_DPPH_ was found in pine buds extracts compared with poplar buds, birch buds and propolis extracts (*p* < 0.05).

### 2.4. SPF Factor of Extracts

**Figure 4:**
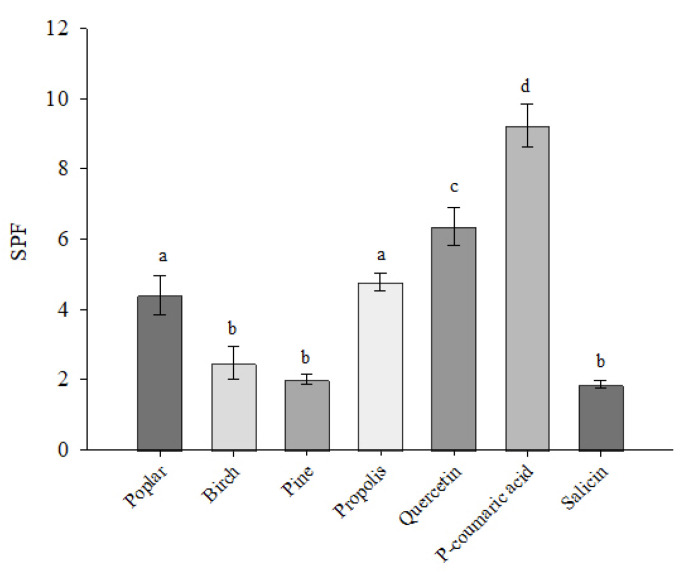
SPF values of poplar, birch, pine buds and propolis extracts by spectrophotometric method. Results are presented at extract concentrations of 10 µg CAE/mL, standards of quercetin, *p*-coumaric acid and salicin at 10 µg/mL. Subscripts of different letters symbolize a statistically significant difference between samples (*p* < 0.05).

The sun protection factor (SPF) of the ethanolic extracts studied was assessed spectrophotometrically (Figure 4). The results showed that the SPF of 10 µg/mL extracts ranged from 2.010 to 4.851. The highest SPF was found in the propolis extract at 4.851. The lowest SPF was found in the pine buds extract at 2.010. The SPF values of the standard quercetin, *p*-coumaric acid and salicin were evaluated, and the highest SPF was found for *p*-coumaric acid at 8.921. The *p*-coumaric acid-dominated extracts of poplar buds and propolis showed almost twice the SPF compared with birch and pine buds extracts. *P*-coumaric acid had a higher SPF compared with quercetin.

### 2.5. Correlation

A significant relation is observed between total phenolic compounds and antioxidant activity (Table 3). A strong negative correlation was observed between total phenolic compounds content and antioxidant activity (IC50_ABTS_ r = −0.870; IC50_DPPH_ r = −0.828). A strong correlation is observed between the total amount of phenolic compounds and SPF values (r = 0.845). Additionally, a strong correlation was found between the total amount of identified phenolic acids and SPF values (r = 0.962).

## 3. Discussion

Propolis and its extracts are used for medicinal purposes. The results of our study confirmed that the chemical composition of propolis extracts depends on the plants from which the bees collect resins for propolis [4,31,32,33]. The *p*-coumaric acid was the predominant acid in the extracts of Lithuanian propolis [34,35]. Socha et al. studied propolis ethanolic extract from different regions of Poland and found *p*-coumaric acid to be the predominant phenolic acid, ranging from 37.54 to 116.95 mg/g, and high levels of ferulic acid [36]. The methanolic extract samples of Turkish propolis also contained *p*-coumaric acid and ferulic acid [37]. The results of this study show that *p*-coumaric acid is predominant in poplar buds extracts, with lower levels found in birch and pine buds extracts. Ferulic acid was also found in the propolis extracts studied and accounted for approximately 25% of the total compounds identified. Ferulic acid, which is one of the main acids in propolis, was not identified in the poplar and birch extracts, while small amounts were detected in the pine buds extracts. *P*-coumaric, chlorogenic and caffeic acids were the most abundant compounds identified in brown and green Brazilian propolis [38]. In our experimental studies, caffeic acid was not identified only in birch buds extracts. Chlorogenic acid was identified only in poplar buds extracts. Cinnamic acid was found in abundance in the poplar buds extracts, accounting for about 29% of the total compounds identified. Small amounts of cinnamic acid were found in extracts of propolis, birch and pine buds, whose amounts are 0.191 ± 0.009 mg/g, 0.097 ± 0.009 mg/g and 0.015 ± 0.001 mg/g, respectively.

The flavonoids identified and quantified in the extracts studied are important for their antioxidant and anti-inflammatory properties. A different range of flavonoids was found in the extracts studied. Quercetin and kaempferol were identified in birch buds extracts at 0.53 ± 0.06 mg/g and 1.53 ± 0.08 mg/g, respectively, and traces of these compounds were detected in pine buds extracts. Samples of methanolic extracts of Turkish propolis contained high levels of quercetin, a potent antioxidant [37]. Quercetin was not identified in the propolis and poplar buds extracts we analyzed, and small amounts were detected in birch and pine buds extracts. Galangin was identified only in the poplar buds extracts and accounted for about 21% of the total compounds identified in the extract. Szliszka et al. found that the flavonoids pinobansin, chrysin and methoxyflavonin were the predominant flavonoids in Polish propolis extract [39]. In Lithuanian propolis extracts, vanillin was the predominant phenolic aldehyde, and smaller amounts of flavonoids pinocembrin, pinobanksin and apigenin were also identified. Vanillin was identified only in propolis extracts. Pinocembrin and pinobanksin were identified in all extracts tested. The highest levels of pinocembrin were found in birch buds extracts. Lower amounts of pinocembrin were found in pine, poplar buds and propolis extracts (0.153 ± 0.005 mg/g, 1.263 ± 0.049 mg/g and 0.509 ± 0.030 mg/g, respectively). Salicin was found in poplar buds extracts and has important pharmacological effects in the treatment of fever, pain and inflammation [40]. Although poplar is one of the plant precursors of propolis, this compound has not been identified in propolis extracts. For most cases analyzed in the scientific literature, propolis is of mixed origin. Often, remnants from several plant precursors can be detected in its composition [16]. The results of the studies have confirmed that the chemical composition of propolis is closely linked to the dominant vegetation in the area of propolis collection [35,41].

The identified active compounds and their levels in the extracts of propolis and its plant precursors have important implications for their biological activity. *P*-coumaric acid, which is strongly predominant in propolis and poplar buds extracts, is known as a hydroxyl compound of cinnamic acid, which is able to reduce the peroxidation of low-density lipoproteins, has antimicrobial activity, contributes to the inhibition of cellular melanogenesis, and is known to have a positive effect on the regulation of the human immune system [42]. Reactive oxygen species (ROS) are produced by normal physiological stresses and cause a variety of cellular damages, which may lead to the accumulation of lipid peroxides in biological membranes [43]. ROS can damage biomolecules that are essential for the body, such as nucleic acids and carbohydrates, as well as affecting and damaging DNA, leading to mutations [44]. If ROS are not effectively removed from cellular components, they can lead to free radical chain reactions that damage biomolecules, resulting in various diseases and ailments. It has long been described in the scientific literature that ROS-induced oxidative stress is one of the main factors in the development of cataracts and the formation of hydrogen peroxide, which is a major intracellular ROS that can activate a wide range of signaling events, promote apoptosis of lens epithelial cells (HLE), and induce lens opacification, which can subsequently lead to the development of cataracts [45]. The adverse effects of UVB-activated ROS on DNA, proteins and lipids are also frequently addressed in the scientific literature [46,47]. In studies by An et al., a strong antimelanogenic effect of *p*-coumaric acid was observed in human epidermal melanocytes exposed to UVB. The study showed that *p*-coumaric acid is a potent and selective inhibitor of human TYR and may be useful as a hypopigmenting agent [48]. In an experimental study, it was found that all extracts exhibited antioxidant activity as assessed by DPPH and ABTS methods in vitro. Pine buds were found to have the highest IC50 concentration by both methods. Propolis and poplar buds extracts showed the lowest IC50 concentrations of 49.92 ± 5.71 µg CAE/mL, 50.89 ± 1.46 µg CAE/mL (ABTS) and 51.92 ± 4.95 µg CAE/mL, 50.64 ± 6.49 µg CAE/mL (DPPH), respectively. In the antioxidant activity assays performed by Wezgowiec et al., the IC50_DPPH_ of the ethanolic propolis extract was found to range from 33.01 ± 2.73 µg/mL to 78.02 ± 4.86 µg/mL by different regions of the collected samples [49]. The results obtained correlate directly with the total phenolic compounds found in the extracts. In a study by Ahn et al., *p*-coumaric acid showed lower antiradical activity against DPPH and reducing power than the flavonoids quercetin or kaempferol [50].

Researchers are focusing on the ability of plant extracts to protect against the UV radiation that causes skin damage. Propolis has been shown to have anticancer [51,52,53,54], anti-inflammatory [55,56,57,58], antioxidant [59,60,61,62] and mainly antibacterial properties [63,64,65,66,67]. Quercetin, a flavonoid with well-known antioxidant activity [68,69], has been used as a reference antioxidant compound for the evaluation of the activity of different extracts. The results of our studies show that *p*-coumaric acid has a stronger SPF value compared with quercetin. The assessment of the SPF of the extracts showed that the extracts of propolis and poplar buds, which are dominated by *p*-coumaric acid, have the best effect. Our results show a correlation between the phenolic acids content and the SPF values of the extracts. The results show that the SPF increased accordingly with increasing concentrations of the identified active compounds in the extracts. The FDA (Food and Drug Administration of the United States and the European Union) recommends the use of ingredients with SPF values above 15 in pharmaceutical products [70].

## 4. Materials and Methods

### 4.1. Materials

Standard solvents and reagents used were of analytical grade. We produced 96% rectified ethanol (JSC “Vilniaus degtine”, Vilnius, Lithuania) and purified deionized water using water purification system Milli-Q^®^ (Millipore, Burlington, MA, USA). Acetonitrile (Sigma-Aldrich, Steinheim, Germany), Folin-Ciocalteu’s reagent (Sigma-Aldrich, St. Louis, MO, USA) and sodium carbonate (Sigma-Aldrich, Saint-Quentin-Fallavier, France). Reference standards: *p*-coumaric acid, cinnamic acid, caffeic acid, vanillin, apigenin and chlorogenic acid (Sigma-Aldrich, Steinheim, Germany); salicin, pinobanksin, pinocembrin, galangin, ferulic acid and vanillic acid (Sigma-Aldrich, St. Louis, MO, USA). ABTS (2,20-azino-bis (3- ethylbenzothiazoline-6-sulfonic acid) (Sigma-Aldrich Chemie, Steinheim, Germany), DPPH (2,2-diphenyl-1-picrylhydrazyl) (Sigma-Aldrich, St. Louis, MO, USA). Ultrasonic bath (Bandelin electronic GmbH & Co.KG, Berlin, Germany).

### 4.2. Extraction

Dried balsam poplar buds, birch buds and pine buds were purchased commercially from Jadvyga Balvociute’s organic herb farm. The raw material was collected in the spring of 2022. Propolis was commercially purchased from R. Serksniene’s farm, in Lithuania, Raseiniai district. We used 70% ethanol (*v*/*v*) as extractant. The ratio of raw material and extractant was 1:10. The extraction was carried out using maceration. Macerates are stored in a dark glass bottle for 7 days at room temperature (21 ± 1 °C), mixing the contents of the macerate several times. The obtained extracts filtered through ashless filter paper.

### 4.3. Evaluation of Total Phenolic Compounds

The total phenolic content of balsam poplar buds, birch buds, pine buds and propolis extracts was evaluated according to Singleton et al. methodology with certain modifications [71]. The phenolic compounds content was determined using the Folin–Ciocalteu reagent. The reaction was carried out in 25 mL volumetric flasks: 1 mL of the test extract, 9 mL of purified water and 1 mL of Folin–Ciocalteu reagent were added and mixed, and after 2–3 min, 1.5 mL of (7.5%) Na_2_CO_3_ was added. The reaction mixtures were diluted with purified water to the mark of 25 mL. Samples were incubated for 40 min at room temperature (21 ± 1 °C) in the dark. The absorbance was measured using a spectrophotometer (Ag-ilent Technologies 8453 UV-Vis, Santa Clara, California, USA) at a wavelength of 760 nm. The results expressed as mg of *p*-coumaric acid equivalent/g of dry weight (mg CAE/g, DW).

### 4.4. HPLC Analysis

Analysis of the phenolic compounds of the extracts under study performed using high-performance liquid chromatography (HPLC). Chromatographic system “Waters 2695” with diode matrix detector “Waters 996” and chromatographic column ACE 5C18, 250 × 4.6 mm data are processed by Empower 2 Chromatography Data software. HPLC eluents consisted of acetonitrile and trifluoroacetic acid. Column temperature 25 °C, flow time 81 min, injection volume 10 µL, mobile phase flow rate 1 mL/min. The compounds present in the sample were identified by the retention time of analytes and reference materials and UV absorption (250 to 400 nm). Reference compounds: salicin (R^2^ = 0.9999), *p*-coumaric acid (R^2^ = 0.9999), cinnamic acid (R^2^ = 0.9999), caffeic acid (R^2^ = 0.9999), chlorogenic acid (R^2^ = 0.9999), pinocembrin (R^2^ = 0.9998), pinobanksin (R^2^ = 0.9999), vanillin (R^2^ = 0.9999), vanillic acid (R^2^ = 0.9999), ferulic acid (R^2^ = 0.9999), neichlorogenic acid (R^2^ = 0.9999), kaempferol (R^2^ = 0.9999), quercetin (R^2^ = 0.9999), apigenin (R^2^ = 0.9998) and galangin (R^2^ = 0.9999).

### 4.5. Antioxidant Activity by DPPH and ABTS Methods In Vitro

The antioxidant activity of extracts ABTS and DPPH in vitro was determined based on the methodology described by Rezzoug et.al with certain modifications [72].

ABTS primary solution was prepared (0.0548 g of ABTS is dissolved in 50 mL of purified water, 0.0095 g of K_2_S_2_O_8_). The prepared ABTS solution was kept in the dark for about 16 h. A working solution of ABTS•+ was prepared by diluting the original solution with purified water until the absorbance of 10 mm solution reaches 0.8 ± 0.03 at 734 nm wavelength. We mixed 50 µl of extract samples (from 1 µg CAE/mL to 100 µg CAE/mL) with 1450 µl of working ABTS•+ solution. The samples were incubated in the dark for 10 min and the absorbance of the samples was measured. Purified water was used as a blank.

For evaluation of DPPH antioxidant activity, a 60 µM DPPH solution in 96% ethanol (*v/v)* was prepared. WE mixed 1 mL of extracts samples (from 1 µg CAE/mL to 100 µg CAE/mL) with 2 mL of DPPH solution. The samples were incubated in the dark for 30 min and their absorbance was measured at 517 nm wavelength. We used 96% ethanol (*v*/*v*) as a blank.

The antiradical activity (%) was estimated based on the formula [73]:

Scavenging effect (%) =((A1 − A2)/A1) × 100(1)

A1 represents absorption of DPPH or ABTS plus blank respectively; A2 represents absorbance of DPPH or ABTS radical with the test sample. The antioxidant activity of the test samples was expressed as an IC50 value.

### 4.6. SPF Factor of Extracts

The sun protection factor of balsam poplar buds, birch buds, pine buds and propolis extracts was evaluated according to Oliveira-Júnior et al. methodology with certain modifications [74]. During the study, the extracts were diluted with 96% ethanol *(v*/*v*) to a concentration of 10 µg CAE/mL. The absorption spectrum of the test samples was obtained in the range of 290–450 nm. A 1 cm quartz element was used for the study. Absorbance data were obtained from 290 to 320 nm in 5 nm increments. We used 96% ethanol *(v*/*v*) as a blank.

Sun protection factor (SPF) spectrophotometric results were calculated according to Mansur et al. equation [75]:

(2) $$\mathrm{SPF}=\mathrm{CF}\;\times \sum_{290}^{320}\mathrm{EE}\;(\lambda )\times I\;(\lambda )\times \mathrm{Abs}\;(\lambda )$$

EE (λ)—erythemal effect spectrum; I (λ)—solar intensity spectrum; Abs (λ)—absorbance of extract; CF—correction factor (= 10).

The constants for the product of the erythemal effect and the intensity of the solar spectrum were estimated by Sayre et al. and are presented in Table 4 [76].

### 4.7. Statistical Analysis

Results expressed as mean and standard deviation of three measurements. One-way ANOVA was used to determine the differences between the compared data that were statistically significant. Tukey’s multiple comparison test was applied. The differences evaluated as statistically significant at *p* < 0.05. Pearson correlation coefficient was determined to evaluate the data correlation (0.3 < |r| < 0.5, weak correlation; 0.5 < |r| < 0.7, medium correlation; 0.7 < |r| < 0.9, strong correlation; 0.9 < |r| ≤ 1, very strong correlation). Data processed and graphically presented using IBM SPSS Statistics 27 (SPSS Inc., Chicago, IL, USA) and SigmaPlot 13.0 (Systat Software, San Jose, CA, USA).

## 5. Conclusions

Buds of poplar, birch and pine growing in Lithuania are characterized by a different composition of active compounds and their quantities. The results of the research allow us to state that the range of active compounds prevailing in Lithuanian propolis extracts is close to birch and poplar buds extracts. Balsam poplar buds extracts and propolis extracts have a higher amount of tested compounds compared with birch and pine buds extracts. The results of this study provide new data on the SPF value of the extracts. The results of antioxidant activity and SPF values correlated with the determined amount of phenolic compounds in the extracts.

## Figures and Tables

**Table 1:** Poplar buds, birch buds, pine buds and propolis total amount of phenolic compounds expressed as mg CAE/g dry weight (DW). Results are presented as mean and standard deviation of three measurements. Different subscript letters indicate a statistically significant difference between samples (*p* < 0.05).

Extracts	mg CAE/g ± SD
1. *Populus balsamifera* buds	230.42 ± 14.83 ^a^
2. *Betula pendula* buds	144.98 ± 10.77 ^b^
3. *Pinus sylvestris* buds	53.93 ± 3.24 ^c^
4. Propolis	222.41 ± 11.29 ^a^

**Table 2:** HPLC analysis of ethanolic poplar, birch, pine buds and propolis extracts (mg/g). Results expressed as mean and standard deviation (SD) of three measurements.

	Poplar mg/g	Birch mg/g	Pine mg/g	Propolis mg/g
Salicin	0.556 ± 0.046	-	-	-
Chlorogenic acid	0.051 ± 0.005	-	-	-
Apigenin	0.792 ± 0.065	1.030 ± 0.065	-	0.370 ± 0.037
Caffeic acid	0.828 ± 0.040	-	0.006 ± 0.0005	0.428 ± 0.020
*P*-coumaric acid	12.696 ± 0.366	1.167 ± 0.078	0.084 ± 0.010	15.776 ± 0.410
Cinnamic acid	8.866 ± 0.167	0.097 ± 0.009	0.015 ± 0.001	0.191 ± 0.009
Pinobanksin	0.314 ± 0.025	0.194 ± 0.012	0.009 ± 0.001	0.315 ± 0.017
Pinocembrin	1.263 ± 0.049	4.940 ± 0.125	0.153 ± 0.005	0.509 ± 0.030
Galangin	6.396 ± 0.110	-	-	-
Vanillin	-	-	-	4.980 ± 0.163
Vanillic acid	-	-	0.010 ± 0.001	0.176 ± 0.017
Ferulic acid	-	-	0.069 ± 0.002	7.479 ± 0.227
Neochlorogenic acid	-	0.078 ± 0.007	-	-
Quercetin	-	0.525 ± 0.063	0.010 ± 0.0003	-
Kaempferol	-	1.526 ± 0.076	0.016 ± 0.001	-
	**Total Phenolic Acids, mg/g**	**Total Flavonoids, mg/g**	**Total Identified Active Compounds, mg/g**	
**Poplar**	22.441	8.765	31.762	
**Birch**	1.342	8.215	9.557	
**Pine**	0.184	0.188	0.372	
**Propolis**	24.050	1.194	30.224	

**Table 3:** Correlation table comparing SPF factor (SPF), antioxidant activity (IC50_ABTS_ and IC50_DPPH_), total phenolic compounds (TPC), total identified phenolic acids (sum_TPA), total identified flavonoids (sum_F) and total identified active compounds (Total_IAC).

		SPF	IC50_ABTS_	IC50_DPPH_	TPC	Sum_TPA	Sum_F	Total_IAC
**SPF**	Pearson Correlation	--						
**IC50_ABTS_**	Pearson Correlation	−0.842 **	--					
	Sig. (2-tailed)	0.001						
**IC50_DPPH_**	Pearson Correlation	−0.712 **	0.928 **	--				
	Sig. (2-tailed)	0.009	0.000					
**TPC**	Pearson Correlation	0.845 **	−0.870 **	−0.828 **	--			
	Sig. (2-tailed)	0.001	0.000	0.001				
**Sum_TPA**	Pearson Correlation	0.962 **	−0.807 **	−0.666 *	0.863 **	--		
	Sig. (2-tailed)	0.000	0.001	0.018	0.000			
**Sum_F**	Pearson Correlation	0.124	−0.572	−0.596 *	0.371	0.091	--	
	Sig. (2-tailed)	0.069	0.052	0.041	0.235	0.779		
**Total_** **IAC**	Pearson Correlation	0.953 **	−0.911 **	−0.802 **	0.914 **	0.974 **	0.299	--
	Sig. (2-tailed)	0.000	0.000	0.002	0.000	0.000	0.345	

**. Correlation is significant at the 0.01 level (2-tailed). *. Correlation is significant at the 0.05 level (2-tailed).

**Table 4:** Normalized values of erythema effect and solar spectrum intensity [76].

	Wavelength (λ)	EE × I (Normalized)
1	290	0.015
2	295	0.0817
3	300	0.2874
4	305	0.3278
5	310	0.1864
6	315	0.0839
7	320	0.018
Total		1

## Data Availability

All data are available in a publicly accessible repository.
